# 2-Methyl-3-phenyl­sulfonyl-5-propyl-1-benzofuran

**DOI:** 10.1107/S1600536808025786

**Published:** 2008-08-16

**Authors:** Hong Dae Choi, Pil Ja Seo, Byeng Wha Son, Uk Lee

**Affiliations:** a Department of Chemistry, Dongeui University, San 24 Kaya-dong, Busanjin-gu, Busan 614-714, Republic of Korea; b Department of Chemistry, Pukyong National University, 599-1 Daeyeon 3-dong, Nam-gu, Busan 608-737, Republic of Korea

## Abstract

The title compound, C_18_H_18_O_3_S, was prepared by the oxidation of 2-methyl-3-phenyl­sulfanyl-5-propyl-1-benzofuran with 3-chloro­peroxy­benzoic acid. The phenyl ring makes a dihedral angle of 81.74 (6)° with the plane of the benzofuran fragment. The crystal structure is stabilized by C—H⋯π inter­actions between a methyl H atom and the phenyl ring of the phenyl­sulfonyl substituent from a neighbouring mol­ecule, and by inter­molecular C—H⋯O inter­actions.

## Related literature

For the crystal structures of similar 2-methyl-3-phenyl­sulfonyl-1-benzofuran compounds, see: Choi *et al.* (2008*a* ▶,*b* ▶).
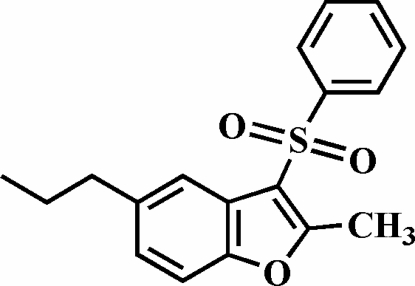


## Experimental

### 

#### Crystal data

C_18_H_18_O_3_S
*M* *_r_* = 314.38
Monoclinic, 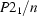

*a* = 7.2712 (9) Å
*b* = 17.583 (2) Å
*c* = 12.788 (2) Å
β = 102.669 (2)°
*V* = 1595.1 (4) Å^3^
*Z* = 4
Mo *K*α radiation
μ = 0.21 mm^−1^
*T* = 173 (2) K
0.40 × 0.40 × 0.30 mm

#### Data collection

Bruker SMART CCD diffractometer
Absorption correction: none
8966 measured reflections
3125 independent reflections
2606 reflections with *I* > 2σ(*I*)
*R*_int_ = 0.053

#### Refinement

*R*[*F* ^2^ > 2σ(*F* ^2^)] = 0.045
*wR*(*F* ^2^) = 0.135
*S* = 1.12
3125 reflections
200 parameters
H-atom parameters constrained
Δρ_max_ = 0.36 e Å^−3^
Δρ_min_ = −0.37 e Å^−3^

### 

Data collection: *SMART* (Bruker, 2001 ▶); cell refinement: *SAINT* (Bruker, 2001 ▶); data reduction: *SAINT*; program(s) used to solve structure: *SHELXS97* (Sheldrick, 2008 ▶); program(s) used to refine structure: *SHELXL97* (Sheldrick, 2008 ▶); molecular graphics: *ORTEP-3* (Farrugia, 1997 ▶) and *DIAMOND* (Brandenburg, 1998 ▶); software used to prepare material for publication: *SHELXL97*.

## Supplementary Material

Crystal structure: contains datablocks global, I. DOI: 10.1107/S1600536808025786/gw2046sup1.cif

Structure factors: contains datablocks I. DOI: 10.1107/S1600536808025786/gw2046Isup2.hkl

Additional supplementary materials:  crystallographic information; 3D view; checkCIF report

## Figures and Tables

**Table 1:** Hydrogen-bond geometry (Å, °) *Cg* is the centroid of the C9–C14 phenyl ring.

*D*—H⋯*A*	*D*—H	H⋯*A*	*D*⋯*A*	*D*—H⋯*A*
C13—H13⋯O3^i^	0.95	2.60	3.355 (3)	137
C18—H18*C*⋯*Cg*^ii^	0.98	3.29	3.947 (4)	126

Symmetry codes: (i) 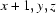; (ii) 
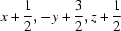
.

**Fig. 1.**
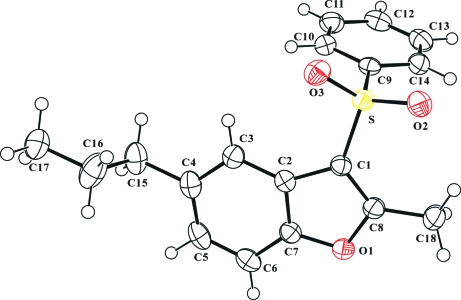
The molecular structure of the title compound, showing displacement ellipsoids drawn at the 50% probability level.

**Fig. 2.**
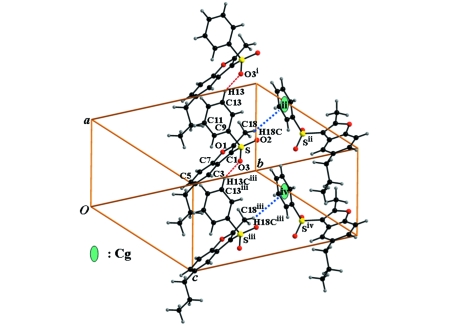
C—H···π and C—H···O interactions (dotted lines) in the title compound. Cg denotes the ring centroid. [Symmetry code: (i) x + 1, y, z; (ii) x + 1/2, -y + 3/2, 1/2 + z+1/2; (iii) x - 1, y, z; (iv) x - 1/2, -y + 3/2, z + 1/2.]

## supplementary crystallographic information

### Comment

This work is related to our communications on the synthesis and structure of 2-methyl-3-phenylsulfonyl-1-benzofuran analogues, *viz*. 5-ethyl-2-methyl-3-phenylsulfonyl-1-benzofuran (Choi *et al.*, 2008*a*) and 5-isopropyl-2-methyl-3-phenylsulfonyl-1-benzofuran (Choi *et al.*, 2008*b*). Here we report the crystal structure of the title compound, 2-methyl-3-phenylsulfonyl-5-propyl-1-benzofuran (Fig. 1).

The benzofuran unit is almost planar, with a mean deviation of 0.018 (2) Å from the least-squares plane defined by the nine constituent atoms. The phenyl ring (C9–C14) makes a dihedral angle of 81.74 (6)° with the plane of the benzofuran fragment. The crystal packing (Fig. 2) is stabilized by intermolecular C—H···π interactions between a methyl H atom and the phenyl ring of the phenylsulfonyl substituent, with a C18—H18C···*Cg*^ii^ separation of 3.291 (4) Å (Fig. 2 and Table 1; *Cg* is the centroid of the C9–C14 phenyl ring, symmetry code as in Fig. 2). The molecular packing is further stabilized by intermolecular C—H···O interactions (Fig. 2 and Table 1; symmetry code as in Fig. 2).

### Experimental

77% 3-Chloroperoxybenzoic acid (471 mg, 2.1 mmol) was added in small portions to a stirred solution of 2-methyl-3-phenylsulfanyl-5-propyl-1-benzofuran (282 mg, 1.0 mmol) in dichloromethane (30 ml) at 273 K. After being stirred for 4 h at room temperature, the mixture was washed with saturated sodium bicarbonate solution and the organic layer was separated, dried over magnesium sulfate, filtered and concentrated under vacuum. The residue was purified by column chromatography (hexane-ethyl acetate, 2:1 *v*/*v*) to afford the title compound as a colorless solid [yield 83%, m.p. 388–389 K; *R*_f_ = 0.75 (hexane–ethyl acetate, 2:1 *v*/*v*)]. Single crystals suitable for X-ray diffraction were prepared by evaporation of a solution of the title compound in acetone at room temperature. Spectroscopic analysis: ^1^H NMR (CDCl_3_, 400 MHz) δ 0.94 (t, J = 7.32 Hz, 3H), 1.62–1.69 (m, 2H), 2,68 (t,J = 7.32 Hz, 2H), 2.79 (s, 3H), 7.12 (d, J = 8.44 Hz, 1H), 7.31 (d, J = 8.44 Hz, 1H), 7.48–7.60 (m, 3H), 7.67 (s, 1H), 8.01 (d, J = 8.44 Hz, 2H); EI-MS 314 [*M*^+^].

### Refinement

All H atoms were positioned geometrically and refined using a riding model, with C—H = 0.95 Å for aromatic H atoms, 0.99 Å for the methylene H atoms, and 0.98 Å for methyl H atoms, respectively, and with *U*_iso_(H) = 1.2Ueq(C) for aromatic and methylene H atoms, and *U*_iso_(H) = 1.5Ueq(C) for methyl H atoms.

### Crystal data

**Table:**

C_18_H_18_O_3_S	*F*_000_ = 664
*M**_r_* = 314.38	*D*_x_ = 1.309 Mg m^−^^3^
Monoclinic, *P*2_1_/*n*	Mo *K*α radiation λ = 0.71073 Å
Hall symbol: -P_2yn	Cell parameters from 3769 reflections
*a* = 7.2712 (9) Å	θ = 2.4–28.3º
*b* = 17.583 (2) Å	µ = 0.21 mm^−^^1^
*c* = 12.788 (2) Å	*T* = 173 (2) K
β = 102.669 (2)º	Block, colourless
*V* = 1595.1 (4) Å^3^	0.40 × 0.40 × 0.30 mm
*Z* = 4	

### Data collection

**Table:**

Bruker SMART CCD diffractometer	3125 independent reflections
Radiation source: fine-focus sealed tube	2606 reflections with *I* > 2σ(*I*)
Monochromator: graphite	*R*_int_ = 0.053
Detector resolution: 10.0 pixels mm^-1^	θ_max_ = 26.0º
*T* = 173(2) K	θ_min_ = 2.0º
φ and ω scans	*h* = −8→5
Absorption correction: none	*k* = −21→21
8966 measured reflections	*l* = −13→15

### Refinement

**Table:**

Refinement on *F*^2^	Secondary atom site location: difference Fourier map
Least-squares matrix: full	Hydrogen site location: inferred from neighbouring sites
*R*[*F*^2^ > 2σ(*F*^2^)] = 0.045	H-atom parameters constrained
*wR*(*F*^2^) = 0.135	*w* = 1/[σ^2^(*F*_o_^2^) + (0.0731*P*)^2^ + 0.5558*P*] where *P* = (*F*_o_^2^ + 2*F*_c_^2^)/3
*S* = 1.12	(Δ/σ)_max_ = 0.001
3125 reflections	Δρ_max_ = 0.36 e Å^−^^3^
200 parameters	Δρ_min_ = −0.37 e Å^−^^3^
Primary atom site location: structure-invariant direct methods	Extinction correction: none

### Special details

**Table:**

Geometry. All e.s.d.'s (except the e.s.d. in the dihedral angle between two l.s. planes) are estimated using the full covariance matrix. The cell e.s.d.'s are taken into account individually in the estimation of e.s.d.'s in distances, angles and torsion angles; correlations between e.s.d.'s in cell parameters are only used when they are defined by crystal symmetry. An approximate (isotropic) treatment of cell e.s.d.'s is used for estimating e.s.d.'s involving l.s. planes.
Refinement. Refinement of *F*^2^ against ALL reflections. The weighted *R*-factor *wR* and goodness of fit *S* are based on *F*^2^, conventional *R*-factors *R* are based on *F*, with *F* set to zero for negative *F*^2^. The threshold expression of *F*^2^ > σ(*F*^2^) is used only for calculating *R*-factors(gt) *etc*. and is not relevant to the choice of reflections for refinement. *R*-factors based on *F*^2^ are statistically about twice as large as those based on *F*, and *R*- factors based on ALL data will be even larger.

### Fractional atomic coordinates and isotropic or equivalent isotropic displacement parameters (Å^2^)

**Table:**

	*x*	*y*	*z*	*U*_iso_*/*U*_eq_	
S	0.61553 (7)	0.71791 (3)	0.30885 (4)	0.02750 (18)	
O1	0.7452 (2)	0.52071 (8)	0.44838 (12)	0.0335 (4)	
O2	0.7324 (2)	0.76700 (8)	0.38556 (12)	0.0367 (4)	
O3	0.4196 (2)	0.73668 (9)	0.27128 (12)	0.0359 (4)	
C1	0.6276 (3)	0.62641 (11)	0.36076 (15)	0.0258 (4)	
C2	0.4937 (3)	0.56569 (11)	0.32516 (16)	0.0266 (4)	
C3	0.3214 (3)	0.55783 (13)	0.25182 (16)	0.0310 (5)	
H3	0.2625	0.6007	0.2131	0.037*	
C4	0.2374 (3)	0.48657 (13)	0.23632 (17)	0.0347 (5)	
C5	0.3257 (3)	0.42403 (13)	0.29533 (19)	0.0403 (6)	
H5	0.2669	0.3756	0.2839	0.048*	
C6	0.4948 (3)	0.43051 (12)	0.36937 (19)	0.0380 (5)	
H6	0.5528	0.3880	0.4094	0.046*	
C7	0.5749 (3)	0.50211 (12)	0.38205 (16)	0.0300 (5)	
C8	0.7750 (3)	0.59641 (12)	0.43314 (16)	0.0304 (5)	
C9	0.7172 (3)	0.71003 (11)	0.19608 (16)	0.0276 (4)	
C10	0.6070 (3)	0.68438 (12)	0.09947 (17)	0.0327 (5)	
H10	0.4781	0.6717	0.0939	0.039*	
C11	0.6880 (4)	0.67762 (13)	0.01194 (18)	0.0403 (6)	
H11	0.6144	0.6603	−0.0546	0.048*	
C12	0.8757 (4)	0.69593 (13)	0.0204 (2)	0.0407 (6)	
H12	0.9304	0.6910	−0.0402	0.049*	
C13	0.9842 (3)	0.72143 (13)	0.1167 (2)	0.0393 (5)	
H13	1.1129	0.7341	0.1218	0.047*	
C14	0.9063 (3)	0.72851 (12)	0.20576 (18)	0.0339 (5)	
H14	0.9804	0.7457	0.2722	0.041*	
C15	0.0537 (3)	0.47675 (16)	0.1550 (2)	0.0445 (6)	
H15A	0.0473	0.5158	0.0986	0.053*	
H15B	0.0549	0.4264	0.1205	0.053*	
C16	−0.1198 (4)	0.4823 (2)	0.1979 (2)	0.0575 (8)	
H16A	−0.1251	0.5334	0.2296	0.069*	
H16B	−0.1129	0.4444	0.2558	0.069*	
C17	−0.2983 (3)	0.46938 (15)	0.1141 (2)	0.0463 (6)	
H17A	−0.2957	0.4185	0.0833	0.056*	
H17B	−0.3080	0.5076	0.0573	0.056*	
H17C	−0.4074	0.4737	0.1472	0.056*	
C18	0.9566 (3)	0.62697 (14)	0.4949 (2)	0.0458 (6)	
H18A	1.0596	0.6087	0.4633	0.069*	
H18B	0.9770	0.6097	0.5695	0.069*	
H18C	0.9532	0.6827	0.4929	0.069*	

### Atomic displacement parameters (Å^2^)

**Table:**

	*U*^11^	*U*^22^	*U*^33^	*U*^12^	*U*^13^	*U*^23^
S	0.0294 (3)	0.0218 (3)	0.0307 (3)	0.00328 (19)	0.0053 (2)	−0.00131 (19)
O1	0.0344 (8)	0.0271 (7)	0.0364 (8)	0.0002 (6)	0.0022 (6)	0.0050 (6)
O2	0.0446 (9)	0.0263 (7)	0.0377 (8)	−0.0017 (7)	0.0059 (7)	−0.0062 (6)
O3	0.0323 (9)	0.0338 (8)	0.0409 (9)	0.0094 (7)	0.0069 (7)	0.0003 (7)
C1	0.0277 (10)	0.0236 (9)	0.0264 (10)	0.0004 (8)	0.0063 (8)	−0.0003 (8)
C2	0.0277 (10)	0.0266 (10)	0.0274 (10)	−0.0005 (8)	0.0099 (8)	−0.0029 (8)
C3	0.0278 (11)	0.0360 (11)	0.0302 (11)	0.0009 (9)	0.0088 (8)	−0.0021 (9)
C4	0.0314 (12)	0.0418 (12)	0.0340 (11)	−0.0063 (9)	0.0143 (9)	−0.0110 (9)
C5	0.0435 (14)	0.0326 (11)	0.0490 (14)	−0.0125 (10)	0.0193 (11)	−0.0104 (10)
C6	0.0452 (14)	0.0271 (11)	0.0440 (13)	−0.0023 (10)	0.0148 (11)	0.0019 (9)
C7	0.0314 (11)	0.0293 (10)	0.0302 (10)	−0.0010 (8)	0.0088 (9)	−0.0002 (8)
C8	0.0327 (11)	0.0259 (10)	0.0312 (10)	0.0002 (9)	0.0042 (9)	0.0008 (8)
C9	0.0314 (11)	0.0202 (9)	0.0309 (11)	0.0037 (8)	0.0065 (8)	0.0043 (8)
C10	0.0315 (11)	0.0325 (11)	0.0327 (11)	−0.0003 (9)	0.0040 (9)	0.0041 (9)
C11	0.0518 (15)	0.0368 (12)	0.0314 (11)	−0.0026 (11)	0.0074 (10)	0.0025 (9)
C12	0.0534 (15)	0.0316 (11)	0.0427 (13)	0.0031 (11)	0.0228 (11)	0.0062 (10)
C13	0.0343 (12)	0.0323 (11)	0.0545 (14)	0.0007 (9)	0.0165 (11)	0.0067 (10)
C14	0.0324 (12)	0.0280 (10)	0.0399 (12)	−0.0006 (9)	0.0045 (9)	0.0018 (9)
C15	0.0374 (13)	0.0589 (15)	0.0392 (13)	−0.0113 (11)	0.0130 (10)	−0.0187 (11)
C16	0.0352 (14)	0.096 (2)	0.0409 (14)	0.0089 (14)	0.0068 (11)	−0.0107 (14)
C17	0.0322 (13)	0.0517 (15)	0.0532 (15)	0.0029 (11)	0.0057 (11)	−0.0032 (12)
C18	0.0391 (13)	0.0368 (12)	0.0523 (14)	−0.0034 (10)	−0.0103 (11)	0.0062 (11)

### Geometric parameters (Å, °)

**Table:**

S—O2	1.437 (2)	C10—C11	1.380 (3)
S—O3	1.438 (2)	C10—H10	0.9500
S—C1	1.735 (2)	C11—C12	1.384 (4)
S—C9	1.765 (2)	C11—H11	0.9500
O1—C8	1.369 (2)	C12—C13	1.384 (4)
O1—C7	1.378 (3)	C12—H12	0.9500
C1—C8	1.360 (3)	C13—C14	1.384 (3)
C1—C2	1.449 (3)	C13—H13	0.9500
C2—C7	1.392 (3)	C14—H14	0.9500
C2—C3	1.398 (3)	C15—C16	1.485 (4)
C3—C4	1.389 (3)	C15—H15A	0.9900
C3—H3	0.9500	C15—H15B	0.9900
C4—C5	1.407 (3)	C16—C17	1.508 (3)
C4—C15	1.512 (3)	C16—H16A	0.9900
C5—C6	1.382 (3)	C16—H16B	0.9900
C5—H5	0.9500	C17—H17A	0.9800
C6—C7	1.382 (3)	C17—H17B	0.9800
C6—H6	0.9500	C17—H17C	0.9800
C8—C18	1.483 (3)	C18—H18A	0.9800
C9—C14	1.391 (3)	C18—H18B	0.9800
C9—C10	1.392 (3)	C18—H18C	0.9800
			
O2—S—O3	118.98 (9)	C10—C11—C12	120.4 (2)
O2—S—C1	108.86 (9)	C10—C11—H11	119.8
O3—S—C1	107.68 (10)	C12—C11—H11	119.8
O2—S—C9	108.30 (10)	C13—C12—C11	120.4 (2)
O3—S—C9	107.87 (9)	C13—C12—H12	119.8
C1—S—C9	104.17 (9)	C11—C12—H12	119.8
C8—O1—C7	106.97 (15)	C12—C13—C14	120.3 (2)
C8—C1—C2	107.64 (18)	C12—C13—H13	119.8
C8—C1—S	125.89 (16)	C14—C13—H13	119.8
C2—C1—S	126.07 (15)	C13—C14—C9	118.7 (2)
C7—C2—C3	119.09 (19)	C13—C14—H14	120.6
C7—C2—C1	104.40 (18)	C9—C14—H14	120.6
C3—C2—C1	136.49 (19)	C16—C15—C4	115.6 (2)
C4—C3—C2	119.1 (2)	C16—C15—H15A	108.4
C4—C3—H3	120.4	C4—C15—H15A	108.4
C2—C3—H3	120.4	C16—C15—H15B	108.4
C3—C4—C5	119.6 (2)	C4—C15—H15B	108.4
C3—C4—C15	119.7 (2)	H15A—C15—H15B	107.4
C5—C4—C15	120.7 (2)	C15—C16—C17	113.4 (2)
C6—C5—C4	122.4 (2)	C15—C16—H16A	108.9
C6—C5—H5	118.8	C17—C16—H16A	108.9
C4—C5—H5	118.8	C15—C16—H16B	108.9
C7—C6—C5	116.4 (2)	C17—C16—H16B	108.9
C7—C6—H6	121.8	H16A—C16—H16B	107.7
C5—C6—H6	121.8	C16—C17—H17A	109.5
O1—C7—C6	126.0 (2)	C16—C17—H17B	109.5
O1—C7—C2	110.60 (18)	H17A—C17—H17B	109.5
C6—C7—C2	123.4 (2)	C16—C17—H17C	109.5
C1—C8—O1	110.39 (18)	H17A—C17—H17C	109.5
C1—C8—C18	134.4 (2)	H17B—C17—H17C	109.5
O1—C8—C18	115.24 (18)	C8—C18—H18A	109.5
C14—C9—C10	121.3 (2)	C8—C18—H18B	109.5
C14—C9—S	119.47 (16)	H18A—C18—H18B	109.5
C10—C9—S	119.19 (17)	C8—C18—H18C	109.5
C11—C10—C9	118.9 (2)	H18A—C18—H18C	109.5
C11—C10—H10	120.6	H18B—C18—H18C	109.5
C9—C10—H10	120.6		
			
O2—S—C1—C8	−27.3 (2)	C1—C2—C7—C6	−177.9 (2)
O3—S—C1—C8	−157.57 (18)	C2—C1—C8—O1	−0.9 (2)
C9—S—C1—C8	88.1 (2)	S—C1—C8—O1	−173.94 (14)
O2—S—C1—C2	160.91 (17)	C2—C1—C8—C18	177.5 (2)
O3—S—C1—C2	30.7 (2)	S—C1—C8—C18	4.4 (4)
C9—S—C1—C2	−83.72 (19)	C7—O1—C8—C1	1.0 (2)
C8—C1—C2—C7	0.4 (2)	C7—O1—C8—C18	−177.68 (19)
S—C1—C2—C7	173.44 (15)	O2—S—C9—C14	19.23 (19)
C8—C1—C2—C3	−177.8 (2)	O3—S—C9—C14	149.23 (16)
S—C1—C2—C3	−4.7 (3)	C1—S—C9—C14	−96.53 (17)
C7—C2—C3—C4	−1.1 (3)	O2—S—C9—C10	−161.66 (16)
C1—C2—C3—C4	176.9 (2)	O3—S—C9—C10	−31.66 (18)
C2—C3—C4—C5	0.7 (3)	C1—S—C9—C10	82.58 (17)
C2—C3—C4—C15	−178.30 (18)	C14—C9—C10—C11	−0.2 (3)
C3—C4—C5—C6	0.1 (3)	S—C9—C10—C11	−179.34 (16)
C15—C4—C5—C6	179.2 (2)	C9—C10—C11—C12	0.1 (3)
C4—C5—C6—C7	−0.6 (3)	C10—C11—C12—C13	−0.2 (3)
C8—O1—C7—C6	177.3 (2)	C11—C12—C13—C14	0.3 (3)
C8—O1—C7—C2	−0.7 (2)	C12—C13—C14—C9	−0.4 (3)
C5—C6—C7—O1	−177.6 (2)	C10—C9—C14—C13	0.4 (3)
C5—C6—C7—C2	0.2 (3)	S—C9—C14—C13	179.49 (16)
C3—C2—C7—O1	178.77 (17)	C3—C4—C15—C16	−94.1 (3)
C1—C2—C7—O1	0.2 (2)	C5—C4—C15—C16	86.9 (3)
C3—C2—C7—C6	0.6 (3)	C4—C15—C16—C17	−178.0 (2)

### Hydrogen-bond geometry (Å, °)

**Table:**

*D*—H···*A*	*D*—H	H···*A*	*D*···*A*	*D*—H···*A*
C13—H13···O3^i^	0.95	2.60	3.355 (3)	137
C18—H18C···Cg^ii^	0.98	3.29	3.947 (4)	126

Symmetry codes: (i) *x*+1, *y*, *z*; (ii) *x*+1/2, −*y*+3/2, *z*+1/2.
